# Grandiose and vulnerable narcissism and regulatory focus at work in relation to strengths use and deficit correction in the workplace

**DOI:** 10.1371/journal.pone.0258609

**Published:** 2021-10-22

**Authors:** Elżbieta Sanecka

**Affiliations:** Institute of Psychology, Faculty of Social Sciences, University of Silesia in Katowice, Katowice, Poland; Aalborg University, DENMARK

## Abstract

Extant research has shown that grandiose narcissism predicts a wide range of work-related outcomes. However, despite differentiating in the social-personality literature besides grandiose narcissism also its vulnerable form, there is little organizational research that would include both expressions of narcissism, in particular with regard to positive organizational behaviors, including strengths use and deficit correction in the workplace. In addition, the role of dark-side personality traits, such as narcissism, in predicting strengths use and deficit correction in the workplace, in particular in regard to motivational factors, seems understudied. Accordingly, this study adds to the literature on narcissism in the organizational context by investigating the direct effects of grandiose and vulnerable narcissism and motivational orientation in the form of regulatory focus at work on strengths use and deficit correction in the workplace. Based on a sample of 446 working adults from the Polish population, it was found that grandiose narcissism positively predicted both strengths use and deficit correction. In turn, vulnerable narcissism was unrelated to strengths use and deficit correction. Furthermore, drawing on the distal-proximal model of work-specific regulatory focus, it also tested whether regulatory focus at work has the incremental validity over grandiose and vulnerable narcissism in predicting strengths use and deficit correction. The hierarchical regression analyses indicated that both promotion and prevention focus were positively associated with strengths use and deficit correction, predicting them beyond grandiose and vulnerable narcissism. The theoretical and practical implications of this study in the context of narcissism in the workplace and positive organizational scholarship are discussed.

## Introduction

Strengths use and deficit correction in the workplace encompass two forms of positive organizational behaviors, which are derived from the strengths-based approach in the positive organizational scholarship. These two separate forms of proactive behavior in the workplace reflect a self-starting and active approach to work goals and tasks. More specifically, strengths use behavior involves the employee taking a personal initiative aimed at using his/her own strengths in the working context, for example by choosing strengths-relevant tasks at work. In contrast, deficit correction behavior entails taking personal initiative as a way to correct own deficiencies, for instance through training or self-development [1].

As prior research demonstrated, both types of proactive organizational behaviors predicted a wide range of positive work-related outcomes, including employee well-being, perception of meaningfulness at work, work engagement, and job performance [1, 2]. However, their relations with personality antecedents are less studied. To date, strengths use and deficit correction behaviors were found to be positively predicted by socially desirable, bright-side individual difference variables, such as proactive personality [1], self-efficacy, psychological capital [3], and core self-evaluations [4]. In turn, the relationships between strengths use and deficit correction and dark personality traits have not yet been examined, despite the well-established, negative associations between socially aversive dispositional constructs and positive organizational behaviors [5–7]. Moreover, it is little known whether some personality traits traditionally termed as “dark”, but demonstrating some brighter sides, such as narcissism [8], might positively predict strengths use and/or deficit correction in the workplace. Indeed, grandiose narcissism, which is widely studied in the organizational context and demonstrated linkages with various behavioral organizational outcomes [9], seems to be particularly important in predicting strengths use and deficit correction in the workplace due to accompanying it tendency to enhance self-image through engaging in positive organizational behaviors [10]. In contrast, as previous research on narcissism, treated as a heterogeneous personality construct, encompassing both grandiosity and vulnerability, and positive organizational behaviors is limited [9, 11–13], the associations between vulnerable expression of narcissism and strengths use and deficit correction seems more unclear and needs additional studies. Therefore, the present study investigated how two major forms of narcissism (i.e., grandiose and vulnerable narcissism) are related to strengths use and deficit correction in the workplace to address these issues, understudied in the literature. In addition, to further extend the nomological network of strengths use and deficit correction at work, the motivational foundations of these organizational behaviors in terms of regulatory focus at work were also examined. In particular, considering the motivational sources of strengths use and deficit correction in the workplace [1, 14] and the role of general regulatory focus in predicting different behavioral outcomes of grandiose and vulnerable narcissism outside the organizational sphere [15, 16], the incremental validity of regulatory focus at work over grandiose and vulnerable narcissism in predicting strengths use and deficit correction was tested.

### Theoretical background

#### Grandiose and vulnerable narcissism

Narcissism is a personality construct rooted in clinical psychology, which successfully penetrated into the subclinical sphere [17, 18]. As a result, in the social-personality and organizational literature, it is generally treated as a continuous variable (a dimensional personality trait) and studied in nonclinical populations, including working adults [9, 19]. In most of these studies, the term narcissism is identified with its grandiose form [20], albeit narcissism, considered as a trait, is recognized as a complex, multidimensional construct on a theoretical level [21, 22].

Reflecting the heterogeneity of the construct, its two main expressions or forms might be distinguished, i.e., grandiose and vulnerable narcissism. In general, grandiose narcissism is mainly characterized by exhibitionism, self-confidence, sense of superiority, dominance, aggression, and social exploitativeness. In turn, vulnerable narcissism is marked by self-inhibition, defensiveness, feeling of inadequacy, anxiety, and negative affect [23–26]. Both dimensions of narcissism share some key characteristics of the construct, among which interpersonal antagonism, entitlement, self-importance, egocentrism, and social selfishness are most often indicated [8, 21, 22, 27]. However, they simultaneously display significant differences in many areas (including their etiology, the self-regulatory mechanisms utilized, and the tendency to be studied in various populations) and have distinct nomological networks [22, 25, 22, 28].

In organizational science, the distinction between grandiose and vulnerable narcissism has been rarely applied, as most studies on narcissism in the work domain examine its grandiose variant [9]. Within these studies, grandiose narcissism is traditionally depicted as a part of the dark-side spectrum of personality in the workplace [7]. As a result of recognizing grandiose narcissism as a prototypical dark personality trait, most previous research on narcissism in the organizational context has sought to identify its negative consequences for the organization itself or for other people in the organization, including a wide range of undesirable, socially maladaptive work-related behaviors and attitudes [29–34]. However, despite the prevalence of studies emphasizing the negative role of narcissism in the workplace, organizational researchers have recently tried to adopt a more nuanced perspective on narcissism in the organizational context. Similarly to the social-personality perspective on narcissism, they tend to describe narcissism in its grandiose form as a “mixed blessing” or “trade-off” [19, 24], stressing not only its negative consequences, but also indicating potential adaptability in the work environment by bringing also some positive implications for the individual and the organization [35–39]. In contrast to the grandiose variant of narcissism, vulnerable narcissism, mainly due to accompanying it increased levels of psychological distress and negative emotionality [25], appears to have less advantageous behavioral and attitudinal manifestations in work settings, including higher emotional exhaustion and lower work engagement [11]. However, single empirical evidence suggests that vulnerable narcissism might also lead to some less detrimental consequences in the organizational context, such as being less prone to gain social attention and boost ego by telling others about own helping behaviors at work [12].

To sum up, although previous studies bring some interesting results in the context of searching for negative and potentially beneficial behavioral consequences of grandiose and vulnerable narcissism in the work environment, this area of organizational research seems still understudied. Therefore, the present study aims to fill this gap in the organizational literature by adopting the above-mentioned prominent perspectives utilized in the studies on narcissism in nonclinical populations. Firstly, in response to the call [9, 11, 29] to address the heterogeneity of narcissism in the studies of the latter in organizational settings, two major forms of narcissism (i.e., grandiose and vulnerable) were included to highlight their unique associations with distinct positive work outcomes. Moreover, in line with the research trend focused on investigating the potentially advantageous aspects of the dark-side personality traits [6, 40], it was examined how both variants of narcissism are related to the constructs derived from the positive organizational scholarship, such as strengths use and deficit correction in the workplace.

#### Grandiose and vulnerable narcissism in relation to strengths use and deficit correction in the workplace

Based on the theoretical and empirical underpinnings, in particular grandiose narcissism was expected to be positively associated with strengths use and deficit correction at work. Theoretically, in terms of the extended agency model of narcissism [41], strengths use and deficit correction behaviors seem to represent self-regulatory strategies used by those high in grandiose narcissism in the workplace in order to manifest and maintain positive, inflated self-beliefs in agentic domains, which–in turn–generate positive feelings about themselves and their abilities. Similarly, as according to the narcissism spectrum model [21], narcissistic grandiosity manifests itself in a bold, exhibitionistic self-regulatory style marked by a self-confident pursuit of agentic goals, such as status, power, and admiration, focusing on using personal strengths and diminishing weaknesses seems to help attain these goals by demonstrating own competence and creating a positive social image among other people in the organization. As a result, those high in grandiose narcissism might apply strengths use and deficit correction both as a way to boost their own positive, inflated self-view by demonstrating that they are the best at their work and–similarly to others forms of positive work behaviors [10]—as an impression management strategy aimed to facilitate their career development in the organization. In addition, their approach orientation and the general pursuit of rewards [21] might lead to finding more opportunities to use strengths and to correct deficits in the workplace. For example, those high in grandiose narcissism might more actively search for professional challenges enabling them to demonstrate their own strong points or participate in additional training and mentoring to show their ability to overcome weaknesses. Prior empirical investigations seem to partially support the assumption that grandiose narcissism might serve as a positive predictor of strengths use and deficit correction at work. In particular, grandiose narcissism was found to be related to proactive organizational behaviors in the form of voice and taking charge [42], subjective career success, engagement in proactive career behaviors and self-reported occupational efficacy [37], suggesting that individuals high in grandiose narcissism are career-oriented, convinced about their high abilities in the vocational sphere, and tend to gain recognition and status in the workplace through different active organizational behaviors.

In contrast, the relations between vulnerable narcissism and strengths use and deficit correction might exhibit different patterns. As vulnerable narcissism manifests itself in reactive orientation linked to avoidance and sensitivity to punishment rather than rewards on the theoretical level [21], it is plausible that in the work setting individuals high in this personality trait would be focused mainly on combating potential threats and obstacles inhibiting career development. Therefore, they might be prone to take action to improve their own deficiencies in the work domain to a greater extent resulting in higher levels of deficit correction. Simultaneously, such employees could be uninterested in actively searching for new vocational opportunities enabling them to use their individual strengths in the workplace. Furthermore, as previous research on narcissism in the workplace showed, individuals with higher vulnerable narcissism are less likely to publicly show off their past positive organizational behaviors [12], which might also translate into decreased strengths use motivated by self-serving, impression management purposes.

#### Regulatory focus at work

In organizational research, the regulatory focus theory (RFT) [43, 44] has gained increased attention in the last two decades as a theoretical framework used to describe self-regulatory mechanisms in the workplace [45–47]. In general, RFT posits two independent, orthogonal self-regulatory or motivational systems, which enable the individual to fulfill different survival needs in the course of goal pursuit: promotion focus and prevention focus. Promotion focus serves growth and development needs, as those high in promotion focus are oriented to desired end-states (aspirations and accomplishments) aligned with their ideal selves. Conversely, prevention focus is derived from security and safety needs, and high prevention-focused people tend to avoid incongruities with the desired end-states, acting in accordance with their ought selves in the pursuit of fulfilling duties and responsibilities [45, 48, 49]. At the behavioral level, both regulatory foci display divergent manifestations in various life domains. For instance, promotion focus is expressed in taking into account different alternatives and openness to change, whereas prevention focus manifests itself in meticulously verifying all available options, accepting rules and maintaining the *status quo* [50].

In the organizational context, both self-regulatory strategies have distinct nomological networks with different antecedents and work-related outcomes [46, 49]. Meta-analytic research by Gorman and colleagues [46] has shown that promotion focus is positively associated with bright-side dispositional variables and positive work attitudes and behaviors. In contrast, prevention focus is linked to negatively evaluated individual difference variables and bring negative consequences in the workplace. Further studies showed a more complex view of the relationships between regulatory foci and workplace outcomes, indicating the importance of both self-regulatory strategies in the organizational context [45, 47], as prevention focus demonstrated positive relations with positive organizational behaviors, stemming from the tendency to avoid potential errors and costs in the workplace [49, 51–54]. In line with this approach, in the present study, promotion and prevention foci were treated as complementary motivational orientations that might lead to positive workplace outcomes, such as strengths use and deficit correction.

#### Regulatory focus at work in relation to strengths use and deficit correction in the workplace

To date, the relationships between promotion and prevention focus and strengths use and deficit correction in the workplace have not been investigated. However, previous empirical evidence indicates that both regulatory foci affect different forms of proactive behavior in the work environment. In particular, Petrou and Demerouti [55], in the study on motivational foundations of job crafting, found that promotion focus had a positive relation with expansive job crafting in terms of seeking resources and challenges, whereas prevention focus was positively related to job crafting manifested in self-protection through reducing demands. Similarly, Waterwall [56] recently found positive relations between work promotion focus and taking charge, and between work prevention focus and problem prevention. In accordance with these results, given the conceptual underpinnings of the RFT, it was expected that both regulatory foci would exhibit positive associations with the positive organizational behaviors in the form of strengths use and deficit correction in the workplace.

Given that promotion-focused individuals are growth-oriented and concentrated on accomplishments, aspirations, and rewards, tend to meet maximal standards of performance, and approach the realization of their ideals through goal pursuit [44, 49], they seem to be more prone to focus on their strong points in the workplace which could be seen by them as an opportunity to achieve positive work outcomes. Thus, promotion focus might manifest itself in the workplace in behaviors aimed at using strengths in organizational settings to a greater extent when striving to fulfill vocational aspirations. Simultaneously, those who are promotion-focused could tend to improve their deficits, inhibiting them from achieving the highest possible results in the workplace. Consequently, they are likely to engage in activities such as overcoming personal weaknesses, which could help them to achieve high standards of performance.

In turn, prevention-focused employees are security-oriented, focused on duties, obligations, and responsibilities, and tend to prevent mistakes and avoid negative outcomes [44, 47, 49]. As a result, they might, in particular, engage in deficit correction behavior in the workplace as a way to secure meeting the minimally accepted standards of performance and avert undesirable results when performing in-role tasks. However, they might also try to emphasize their own abilities and draw the attention of others to the areas in which they feel competent, represented by their strong points. Consequently, they could treat strengths use as a more secure behavioral strategy and engage in it more frequently.

#### The current study

Taking into account the above conceptual and empirical considerations, the goal of the present study was twofold. Firstly, this study aimed to examine how grandiose and vulnerable narcissism and work-specific promotion and prevention focus would be related to strength use and deficit correction in the organizational context. Accordingly, the following hypotheses were formulated: (H1) grandiose narcissism would be positively related to strengths use and deficit correction in the workplace, (H2) vulnerable narcissism would be negatively related to strengths use and positively to deficit correction, (H3) promotion and prevention focus would be positively related to strengths use and deficit correction in the workplace. Secondly, when predicting strengths use and deficit correction in the workplace, the current study investigated the incremental predictive validity of regulatory focus at work above and beyond two forms of narcissism.

It is also worth noting that in line with the distal-proximal conceptual framework of regulatory focus at work, which indicates that work-specific regulatory focus better predicts work-related outcomes than general regulatory focus [49], the construct of work-specific regulatory focus was utilized in the present study. In addition, similarly to previous studies on narcissism and regulatory focus [15, 16] and the majority of studies on regulatory focus in the workplace describing it in terms of moderately stable, chronic disposition reflecting individual differences in employees’ needs and values [47, 49, 57, 58], regulatory focus at work was treated as a trait-like (dispositional) variable. It was expected that such an approach would help highlight the relationships between regulatory foci and positive work outcomes in the form of strengths use and deficit correction in the workplace.

## Materials and methods

### Participants and procedure

The current research procedure was approved by the local institutional review board (decision No. KEUS.69/01.2021 of the Ethics Committee of the University of Silesia in Katowice, Poland). All participants completed written informed consent before starting the study.

The survey was preceded by an a priori power analysis performed using the G*Power 3.1.9.4 software [59]. Its results showed that the minimum sample size of 160 individuals was required for the multiple linear regression model with 8 predictors to ensure an error probability of .05, the statistical power of .95, and medium effect size of .15.

The hypotheses were tested on a sample from the general population collected through a web-based survey. To ensure the sample’s representativeness, the study was conducted via an online questionnaire placed on the Polish national research panel (https://www.badanie-opinii.pl/). Research data were collected using the CAWI (computer-assisted web interviewing) methodology between 2 and 4 April 2021. Stratified sampling was applied in order to obtain a representative sample in terms of age and gender from the Polish population of working adults. The individuals registered on the research panel received an invitation to participate in the study on psychological determinants of strengths use in the workplace through e-mail or PUSH notification on their mobile phones. The study was anonymous and voluntary, and those who completed the online research questionnaire received bonus points as compensation for their participation in the study, added to their individual accounts on the research panel; after exceeding the set limit, they could be exchanged into money.

In order to provide high-quality data, the questionnaire started with a short introduction explaining the purpose of the study, stressing its anonymous and voluntary nature, and indicating the practical utility of the obtained results for future strength-based interventions in the organizational context. The participants were also asked to carefully read each question and were informed that there were no wrong or right answers in the study, and only responses reflecting their true feelings or opinions were valuable. Then, they gave informed consent, provided sociodemographic data, and filled in a set of self-reported questionnaires. Of 450 respondents, who completed the survey in a fixed time period and answered to the control questions correctly, one person was excluded due to invalid data (i.e., providing the same answers for different items in each questionnaire). In addition, three influential outliners, identified on the basis of values of Cook`s distance measure, centered leverage values and the Mahalanobis distance measure, were removed from the further analyses.

The final sample was composed of 446 participants, including 251 (56.3%) men and 195 (43.7%) women with age ranging from 18 to 64 (M = 40.24; SD = .60). With regard to education, the majority of respondents had secondary (46.6%, n = 208) or higher (42.6%, n = 190) education, followed by 9.2% (n = 41) with vocational education and 1.6% (n = 7) with elementary education. On average, the participants spent 38.67 hours (SD = .43) working per week, and their organizational tenure varied from less than a year to 48 years (M = 9.10; SD = .40). Most of them were only performing their professional work (88.3%, n = 394), while 9.4% (n = 43) were studying and working, and 2.2.% (n = 10) were retired and working. In terms of the form of employment, employees working under an employment contract for an unspecified (72.2%, n = 322) or a specified (13.9%, n = 69) period predominated, whereas 9.2% (n = 41) were working under a civil law contract of employment, and the remaining 4.7% (n = 21) declared working under other forms of employment. The respondents were working in different organizations varying in size. With regard to the number of employees working for the organization, 28.7% (n = 128) of the respondents were working in large enterprises (having more than 250 employees), 27.8% (n = 124) in medium-sized enterprises (having up to 250 employees), 23.8% (n = 106) in small enterprises (with up to 50 employees), and 19.7% (n = 88) in micro-enterprises (having up to 10 employees). The dataset used in this study is publicly available at the OSF data repository: https://osf.io/nk6a5/.

### Measures

#### Grandiose narcissism

To measure grandiose narcissism, the Narcissistic Personality Inventory (NPI) was used [60, 61]. The Polish validated version of the scale includes 34 items with a 5-point Likert scale ranging from 1 –“it’s not me” to 5 –“it’s me”. Exemplary items are: “I have a natural talent for influencing people” and “I will be a success”. The Polish version of the scale demonstrated good psychometric properties both in the adaptation study [60] and in the previous studies on grandiose narcissism in a nonclinical population [20, 62]. In the present study, Cronbach`s alpha was .94.

#### Vulnerable narcissism

To assess vulnerable narcissism, the Polish version of the Hypersensitive Narcissism Scale (HSNS) was applied [63, 64]. The scale comprises 10 items with a 5-point response format from 1 (“strongly agree”) to 5 (“strongly disagree”). The sample item is “My feelings are easily hurt by ridicule or the slighting remarks of others.” Cronbach’s alpha for the scale was .75.

#### Strengths use and deficit correction in the workplace

To measure strengths use and deficit correction in the workplace, two 6-item subscales form the Strengths Use and Deficit COrrection (SUDCO) questionnaire were used [1]. Participants responded using a 7-point Likert scale from 0 (never) to 6 (almost always). Sample items include: “In my job, I make the most of my strong points” (for strengths use behavior) and “I engage in activities to develop my weak points at work” (for deficit correction behavior). The full instrument, including also the two additional scales (i.e., perceived organizational support for strengths use and perceived organizational support for deficit correction), was translated from English into Polish using the procedure of back-translation. Firstly, three independent forward translations were developed by the three Polish native speakers fluent in English, of whom one was a researcher specializing in work and organizational psychology, and the two others were professional translators, knowing the concept of strengths use and deficit correction in the workplace and the accompanying terminology. Then, the panel of experts, including four work and organizational psychologists (among them the first forward translator), reconciled the final version of the forward translation. In the next step, based on this version of the scale, the independent back translation was carried out by the next professional translator. The comparison of the back-translated scale with the original instrument showed that both versions of the measure are linguistically equivalent. A subsequent principal component analysis conducted for two subscales (i.e., strengths use and deficit correction) indicated two factors, reflecting respectively strengths use and deficit correction in the workplace, which cumulatively accounted for 71.27% of the total variance in the data. The initial eigenvalues amounted to 7.32 for the first factor, and 1.23 for the second factor. The parallel analysis with the oblimin rotation, which was subsequently carried out in Jasp 0.14.1.0. software, confirmed the two-factor solution with both factors after rotation accounting for 66% of the total variance (including 37% for the first factor, and 29% for the second factor). The corrected item-total correlations were satisfactory, ranging from 0.64 to 0.8. Cronbach’s alpha for strengths use behavior was .94, and for deficit correction behavior .89, indicating very good reliability.

#### Regulatory focus in the workplace

The Polish validated version of the Work Regulatory Focus (WRF) Scale was applied to measure work-specific regulatory focus [65, 66]. The scale consists of 18 items rated on a 5-point Likert scale, ranging from 1 (strongly disagree) to 5 (strongly agree). The items are grouped into two subscales, reflecting prevention and promotion focus at work, respectively. Example items included “I concentrate on completing my work tasks correctly to increase my job security” (for prevention focus) and “I tend to take risks at work in order to achieve success” (for promotion focus). Cronbach’s alpha for prevention focus was .88, and for promotion focus .82.

### Controls

Following previous studies on narcissism in the organizational context [11, 32, 67, 68] and strengths use and deficit correction in the workplace [3, 69], age (in years), gender (1 = female, 2 = male), and organizational tenure (in years) were included as control variables. In addition to controlling for the sociodemographic characteristics of participants, given that perceived workload might affect strengths use [70], this job stressor was also incorporated in the present study as a control variable. Workload was assessed by the 5-item Quantitative Workload Inventory (QWI) [71, 72], which is a 5-item instrument developed to measure amount of work. Participants assessed questions referring to perceived quantitative workload (e.g., “How often does your job leave you with little time to get things done?”) on a 5-point Likert-type frequency response scale, ranging from 1 (“less than once per month or never”) to 5 (“several times per day”).

### Statistical analyses

All analyses were performed using the SPSS Statistics package (version 25.0). Firstly, descriptive statistics and bivariate correlations were calculated. To test the hypotheses concerning the direct effects, correlational and multiple hierarchical linear regression analyses were performed.

## Results

### Preliminary analyses

Descriptive statistics and zero-order correlations (Pearson) for all the variables are presented in Table 1.

**Table 1:** Descriptive statistics and bivariate correlations among the study variables.

Variable	M	SD	Range	1.	2.	3.	4.	5.	6.	7.	8.	9.	10.
1. Age	40.24	12.61	18–64	-									
2. Gender	1.56	.50	1–2	.48***	-								
3. Tenure	9.10	8.47	0–43	.60***	.31***	-							
4. Workload	15.47	4.85	5–25	.03	.01	.12*	-						
5. Grandiose narcissism	101.81	21.54	37–167	-.15**	-.03	-.03	.16**	-					
6. Vulnerable narcissism	30.84	5.65	14–48	-.14**	-.06	-.06	.17***	.19***	-				
7. Promotion focus	32.63	5.34	19–45	-.11*	-.12**	-.02	.17***	.47***	.14**	-			
8. Prevention focus	37.19	5.35	18–45	.00	-.17***	.01	.17***	.18***	.09	.56***	-		
9. Strengths use	26.39	7.09	0–36	.06	.03	.06	.10*	.23***	.05	.43***	.53***	-	
10. Deficit correction	23.71	7.03	0–36	-.03	-.05	.01	.17***	.28***	.06	.53***	.46***	.71***	-

*N* = 446. Gender coded: 1 –female, 2 –male.

* *p* < .05.

**p < .01.

***p < .001.

Range = Min-Max.

The results of correlation analysis provided some initial support for hypotheses H1 and H3. There are several significant intercorrelations among the study variables, whose magnitude ranges from small to moderate. As predicted, grandiose narcissism was positively and significantly correlated with strengths use and deficit correction behaviors, but the magnitude of correlations was small. In turn, vulnerable narcissism was not significantly associated with strengths use and deficit correction at the workplace. In line with the expectations, both promotion and prevention foci were moderately positively associated with strengths use and deficit correction. Finally, promotion focus was moderately positively correlated with grandiose, and weakly positively with vulnerable narcissism, while prevention focus was weakly positively correlated with grandiose narcissism.

### Hierarchical regression analyses

Subsequently, two separate three-step hierarchical regression analyses were carried out with strengths use and deficit correction as outcome variables. Control variables (age, gender, organizational tenure, and workload) were entered into the regression models in the first step, grandiose and vulnerable narcissism in the second step, and promotion and prevention focus in the third step. The results of the regression analyses are presented in Table 2.

**Table 2:** The results of hierarchical regression analyses.

Variable	Strengths use		Deficit correction	
Step 1	*B(SE)*	*β*	*B(SE)*	*β*
Age	.03(.04)	.05	-.01(.04)	-.02
Gender	-.10(.77)	-.01	-.72(.76)	-.05
Tenure	.01(.05)	.02	.02(.05)	.02
Workload	.14(.07)	.10*	.24(.07)	.17**
*R*^*2*^	.01		.02	
*F*	1.52		3.54**	
Step 2				
Age	.06(.04)	.11	.02(.04)	.04
Gender	-.28(.75)	-.02	-.93(.73)	-.07
Tenure	.00(.05)	-.00	.00(.05)	.00
Workload	.08(.07)	.06	.18(.07)	.12**
Grandiose narcissism	.08(.02)	.23***	.09(.02)	.27***
Vulnerable narcissism	.01(.06)	.01	-.01(.06)	-.01
*R*^*2*^(Δ *R*^*2*^)	.05 (Δ*R*^*2*^ = .05)		.09 (Δ*R*^*2*^ = .07)	
*F*	5.16***		8.07***	
Step 3				
Age	.02(.03)	.04	.00(.03)	.00
Gender	1.39(.66)	.10*	.44(.65)	.03
Tenure	.01(.04)	.01	.00(.04)	.00
Workload	-.03(.06)	-.02	.08(.06)	.06
Grandiose narcissism	.03(.02)	.09	.02(.02)	.08
Vulnerable narcissism	-.02(.05)	-.01	-.04(.05)	-.03
Promotion focus	.22(.07)	.16**	.46(.07)	.35***
Prevention focus	.58(.07)	.44***	.34(.06)	.26***
*R*^*2*^(Δ *R*^*2*^)	.31 (*ΔR*^*2*^ = .26)		.32 (*ΔR*^*2*^ = .23)	
*F*	26.06***		26.69***	

*N* = 446. Gender coded: 1 –female, 2 –male.

* *p* < .05.

**p < .01.

***p < .001.

For strengths use, the first step with sociodemographic factors and workload entered as control variables were nonsignificant. When the personality variables were included in the regression equation, the regression model became significant. In this model, grandiose narcissism emerged as the only significant predictor of strengths use, explaining a small but significant 5% of additional variance in strengths use. Including regulatory foci in the third step significantly improved the model, accounting for an additional 26% of the unique variance in strength use, and gender, promotion and prevention foci were found as the only significant predictors of strengths use.

For deficit correction, all three steps were significant. In the first step, workload positively predicted deficit correction, accounting for a small, but significant 2% of variance in deficit correction. Results for the second step showed that workload remained a positive predictor of deficit correction, and narcissism was found as a new positive predictor, accounting for an additional 7% of the variance. After the inclusion of regulatory foci in the third step, workload and grandiose narcissism became insignificant. In the final model, only promotion and prevention foci were significant positive predictors, explaining an additional 23% of variance in deficit correction.

In general, results of hierarchical regression analyses demonstrated that only the grandiose form of narcissism significantly positively predicted strengths use and deficit correction, thus fully supporting H1. In contrast, as vulnerable narcissism did not exhibit significant associations with strengths use and deficit correction, H2 was not confirmed. In turn, H3 was fully supported, as both promotion and prevention foci were positively associated with strengths use and deficit correction. The findings also exhibited the incremental predictive validity of regulatory foci of personality traits in the form of grandiose and vulnerable narcissism in relation to the strengths use and deficit correction behaviors. The positive effects of narcissism on both strengths use and deficit correction became nonsignificant after entering two forms of regulatory foci into the regression models, suggesting the mediation role of promotion and prevention focus on the relationship between grandiose narcissism and strengths use and deficit correction.

## Discussion

Narcissistic personality has aroused a lot of interest among organizational researchers in the last two decades, mainly due to its negative consequences at the individual, team, and organizational levels [9, 29, 73]. However, a vast majority of prior studies focused on grandiose narcissism, paying little attention to its vulnerable counterpart. Moreover, there is relatively little research in the organizational literature on the potentially adaptive aspects of narcissism in the workplace. The current study aims at extending this research perspective by investigating how two major forms of narcissism (i.e., grandiose and vulnerable) are associated with positive organizational behaviors represented by strengths use and deficit correction in the workplace. In addition, to broaden the research perspective on individual difference predictors of strengths use and deficit correction, both types of these proactive behaviors at work were examined in relation to motivational orientation in terms of promotion and prevention focus at work. Finally, it was tested whether work-specific regulatory foci predict strengths use and deficit correction behaviors at work over grandiose and vulnerable narcissism.

Supporting the first hypothesis, the results showed that grandiose narcissism was positively associated with both strengths use and deficit correction at work. These findings are in line with previous empirical evidence, demonstrating that grandiose narcissism positively predicted proactive behaviors at work [42]. It is possible that increased levels of self-reported proactive work behaviors among those high in grandiose narcissism might stem from their careerist orientation. Thus, striving for status and power in organizations, they might try to excel in agentic domains by exhibiting strengths use and deficit correction behaviors. Alternatively, such employees are likely to emphasize individual strengths and make additional efforts aimed at diminishing own weaknesses to achieve the impression-management purposes or to nurture grandiose self-view.

Contrary to the predictions, vulnerable narcissism was, in turn, unrelated to strengths use and deficit correction. These results might be explained with regard to behavioral inhibition accompanying narcissistic vulnerability [21]. Highly neurotic, anxiety-driven and shy individuals with high levels of vulnerable narcissism [21, 23] are likely to monitor the work environment searching for potential threats and simultaneously tend to ignore the opportunities to develop own potential through strengths use and deficit correction. As a result, to avoid potential embarrassment or negative evaluation by others at work, they might display the behavioral tendency to passively react to organizational factors rather than take personal initiative in the organization.

The results of this study also indicated that self-regulatory processes play an important role in explaining strengths use and deficit correction behaviors in the workplace, as both types of the analyzed positive work behaviors were positively predicted by work regulatory foci. However, promotion and prevention foci differed in their relationships with strengths use and deficit correction, suggesting that both proactive organizational behaviors might have distinct motivational sources. More specifically, prevention focus was the stronger predictor of strengths use than prevention focus. The inverse pattern of relationships was observed when predicting deficit correction, with prevention focus emerging as the strongest predictor. In addition, as hierarchical regression analyses revealed, promotion and prevention foci predicted strengths use and deficit correction behaviors beyond and above narcissistic grandiosity and vulnerability. More importantly, in predicting promotion strengths use and deficit correction, grandiose narcissism became insignificant after entering them in the regression models in the last step of hierarchical regression analyses. These results suggest that regulatory foci might pose a self-regulatory mechanism underlying strengths use and deficit correction among people with elevated grandiose narcissism. However, in line with the recommendations of Maxwell et al. [74], as the present study relied on a cross-sectional design, to avoid biased results, the mediation effect of regulatory foci on the relationships between grandiose narcissism and two forms of proactive behaviors at work was not tested.

Several strengths of the present study could be delineated. Firstly, this study offers a broader research perspective on organizational narcissism by including its two primary variants, namely grandiose and vulnerable narcissism. Thanks to going beyond the dominant trend in organizational science identifying narcissism only with its grandiose form, the present study might help to better elucidate the differences in behavioral manifestations of distinct expressions of narcissism in the working environment. Moreover, the current study contributes to the organizational literature by utilizing the strength-based approach arising from positive psychology to study narcissism in the work domain. By taking into account the constructs of strengths use and deficit correction, which originate from positive organizational scholarship and are considered as representing positive organizational behaviors, this study highlighted the potentially beneficial, bright-side aspects of narcissism at work. Another strength of this study is the use of data from a representative sample of Polish employees.

Besides bringing the above-mentioned theoretical contributions, the present study offers some practical implications. In particular, examining the motivational foundations of strengths use and deficit correction might help to determine how to encourage employees to engage in positive, pro-organizational behaviors. These findings can be of particular importance for managers and organizational practitioners, planning and introducing positive interventions in organizations. More specifically, the results of this study suggest that future positive psychology interventions at work could benefit from taking into account the motivational factors affecting strengths use and deficit correction among organization members.

### Limitations and future directions

Although this study extends previous literature on narcissism in the organizational context on the one hand, and on strengths use and deficit correction at work on the other, it also has some limitations that warrant discussion. First, the obtained results were based on a single cross-sectional study. As the present study investigates the understudied phenomena in the organizational context, mainly by concentrating on how grandiose and vulnerable narcissism differ in their relationships of with strengths use and deficit correction at work, it was primarily designed to emphasize these differences in the correlation patterns for both expressions of narcissism, and to ascertain the basic relationships between the study variables which have not been examined earlier. Therefore, given the first research phase on the relationships between the study variables, the cross-sectional study design was chosen. However, the replication study using different methods and sources of data and examining the boundary conditions is needed in the future. The reliance on cross-sectional data in the present study prevents from drawing firm conclusions concerning causality. Thus, to better understand how strength use and deficit correction behaviors shape and change over time among those high in narcissism, it is worth to apply a longitudinal study design in the future. As applying the cross-sectional study design excludes the possibility to ascertain the indirect effects [74], collecting the data in two waves in subsequent studies would also help to determine if regulatory foci at work mediate the relationships between grandiose narcissism and strengths use and deficit correction. Furthermore, the exclusive usage of self-report measures might result in a common method bias. To minimalize this concern, the study was designed in such a way as to maximize the participants’ motivation to respond accurately and to reduce task difficulty [75]. For instance, a clear invitation and instruction emphasizing the practical implications and the anonymity of the survey were developed, measures with different response rates were used, and the survey was conducted online to guarantee anonymity for the respondents. However, future research should include more objective organizational criteria (e.g., performance appraisals) or external measures of strengths use and deficit correction at work (e.g., manager- or coworker-ratings) to delineate better the extent of strengths use and deficit correction behaviors. Another possible limitation of this study is that the self-reported levels of strengths use and deficit correction seem to be particularly susceptible to the social desirability bias, in particular in the case of employees high in grandiose narcissism. As grandiose narcissism–especially combined with power–manifests itself in overconfidence [76], highly narcissistic individuals and employees might overestimate their strong points and belittle weaknesses. To avoid overreporting strengths use and underreporting deficit correction by those high in narcissism, subsequent studies should include behavioral measures or other-ratings of these two types of positive organizational behaviors. Alternatively, to ensure greater objectivity of the results, the external job performance indicators could be utilized in the future.

An additional limitation stems from the research period, which might have affected the results concerning the declared strengths use and deficit correction. The present research was carried out during the third wave of the COVID-19 pandemic in Poland in April 2021. The outbreak of the COVID-19 pandemic is considered in the work and organizational literature as a career shock, which had significant short- and long-term consequences for individuals’ work and careers, mainly by generating higher job insecurity [77]. As a result, due to the predominance of contextual factors in the vocational sphere during the COVID-19 crisis, employees–regardless of their level of narcissism–might be more motivated to utilize their own strengths and reduce deficits in order to keep their jobs. Thus, to resolve this doubt, it is worth replicating the findings in a subsequent period after the end of the pandemic.

There are several other possible directions of future research on narcissism within the strengths and weaknesses framework. In particular, as those high in grandiose narcissism are career-oriented and focused on self-promotion [9, 22], they might overuse their strengths in the workplace, leading to the so-called effect of “too much of a good thing”, used to identify the upper limit of beneficial strengths use [78]. Given that both the overuse and underuse of character strengths bring negative consequences for the organization and are considered to reflect psychopathology by some researchers [78, 79], future work should distinguish between distinct aspects of strengths use, treated as a continuous variable, such as the overuse, underuse, and optimal use of strengths. Furthermore, to provide a more complex picture of strengths use and deficit correction in the workplace among individuals high in narcissism and other socially aversive personality traits, subsequent studies need to examine which specific character strengths and to what extent are used by different dark personalities. Another potential avenue of future research includes investigating the role of situational cues (mainly organizational factors) and of situational regulatory focus on the relationships between narcissism and strengths use and deficit behaviors. Finally, given that strengths use may fluctuate and change depending on the contextual variables [80], further studies should apply a diary study or experience sampling method to better understand the dynamic of strengths use and deficit correction in those high in narcissism. Such an approach might help to identify the organizational factors that contribute to activating their proactivity in the workplace.

## Conclusion

In summary, the present research incorporated the strength-based approach in the study on narcissism in the organizational context. Its results demonstrated that grandiose and vulnerable narcissism differ in their relationships with strengths use and deficit correction in the workplace. In particular, only grandiose narcissism predicted strengths use and deficit correction, which suggests that employees high in grandiose narcissism might use these two types of positive organizational behaviors to enhance a positive self-view in the working environment. In contrast, vulnerable narcissism was unrelated to strengths use and deficit correction in the workplace, suggesting that this expression of narcissism manifests in more inhibited behaviors in the workplace. With regard to regulatory foci at work, both promotion and prevention focus positively predicted strengths use and deficit correction at work. In addition, hierarchical regression analyses demonstrated that work-specific regulatory foci accounted for an additional variance above and beyond grandiose and vulnerable narcissism in predicting strengths use and deficit correction. These findings imply that motivational factors might play a decisional role in positive organizational interventions directed towards enhancing proactivity among employees.
